# CNEReg Interprets Ruminant-specific Conserved Non-coding Elements by Developmental Gene Regulatory Network

**DOI:** 10.1016/j.gpb.2022.11.007

**Published:** 2022-12-07

**Authors:** Xiangyu Pan, Zhaoxia Ma, Xinqi Sun, Hui Li, Tingting Zhang, Chen Zhao, Nini Wang, Rasmus Heller, Wing Hung Wong, Wen Wang, Yu Jiang, Yong Wang

**Affiliations:** 1 Key Laboratory of Animal Genetics, Breeding and Reproduction of Shaanxi Province, College of Animal Science and Technology, Northwest A&F University, Yangling 712100, China; 2 Department of Medical Research, Guangdong Provincial People’s Hospital, Guangdong Academy of Medical Sciences, Guangzhou 510080, China; 3 Guangdong Cardiovascular Institute, Guangdong Provincial People’s Hospital, Guangdong Academy of Medical Sciences, Guangzhou 510080, China; 4 Academy of Mathematics and Systems Science, Chinese Academy of Sciences, Beijing 100190, China; 5 School of Mathematics, University of Chinese Academy of Sciences, Beijing 100049, China; 6 State Key Laboratory for Conservation and Utilization of Subtropical Agro-Bioresources, College of Animal Science and Technology, Guangxi University, Nanning 530005, China; 7 Section for Computational and RNA Biology, Department of Biology, University of Copenhagen, Copenhagen DK-2100, Denmark; 8 Department of Statistics, Department of Biomedical Data Science, Bio-X Program, Stanford University, Stanford, CA 94305, USA; 9 Center for Ecological and Environmental Sciences, Northwestern Polytechnical University, Xi’an 710072, China; 10 State Key Laboratory of Genetic Resources and Evolution, Kunming Institute of Zoology, Chinese Academy of Sciences, Kunming 650223, China; 11 Center for Excellence in Animal Evolution and Genetics, Chinese Academy of Sciences, Kunming 650223, China; 12 Key Laboratory of Systems Health Science of Zhejiang Province, School of Life Science, Hangzhou Institute for Advanced Study, University of Chinese Academy of Sciences, Hangzhou 310024, China

**Keywords:** Trait innovation, Gene regulatory network, Conserved non-coding element, Toolkit transcription factor, Ruminant

## Abstract

The genetic information coded in DNA leads to **trait innovation** via a **gene regulatory network** (GRN) in development. Here, we developed a **conserved non-coding element** interpretation method to integrate multi-omics data into gene regulatory network (CNEReg) to investigate the **ruminant** multi-chambered stomach innovation. We generated paired expression and chromatin accessibility data during rumen and esophagus development in sheep, and revealed 1601 active ruminant-specific conserved non-coding elements (active-RSCNEs). To interpret the function of these active-RSCNEs, we defined **toolkit transcription factors** (TTFs) and modeled their regulation on rumen-specific genes via batteries of active-RSCNEs during development. Our developmental GRN revealed 18 TTFs and 313 active-RSCNEs regulating 7 rumen functional modules. Notably, 6 TTFs (OTX1, SOX21, HOXC8, SOX2, TP63, and PPARG), as well as 16 active-RSCNEs, functionally distinguished the rumen from the esophagus. Our study provides a systematic approach to understanding how gene regulation evolves and shapes complex traits by putting evo-devo concepts into practice with developmental multi-omics data.

## Introduction

To answer the key question of how new traits arise during the macroevolutionary process, biologists have long realized the necessity to understand the gene regulation in development responsible for morphological diversity, *i.e.*, which genes are expressed, what regulatory element changes are involved, and how regulatory element changes affect development [1]. Only recently have the field of large-scale omics and the accumulation of data matured sufficiently to explore these theoretical concepts in detail. Here, we investigate the ruminant multi-chambered stomach, a key mammalian organ innovation and a cornerstone of evolutionary theory, as an example to illustrate a novel framework for integrating multi-omics data to address the fundamental question of organ innovation.

The rumen hosts a diverse ecosystem of microorganisms and facilitates efficient plant fiber digestion and short-chain fatty acid uptake, which significantly promotes the expansion and diversification of ruminant animals by providing a unique evolutionary advantage relative to non-ruminants [2]. This remarkable morphological innovation raises the fundamental question of how the genetic toolkit generates functional complexity through development and evolution [1], [3], [4]. By comparing 51 ruminants with 12 mammalian outgroup species genomes, we previously identified 221,166 ruminant-specific conserved non-coding elements (RSCNEs), which span approximately 0.61% of the genome (16.5 Mb in total) [5]. These RSCNEs are potential regulatory elements of proximal or distal genes for transcriptional regulation in the development of morphological and physiological traits [6]. In addition, we previously sequenced two representative ruminants (sheep and roe deer) for gene expression across 50 tissues. Comparative transcriptome analysis revealed 656 rumen-specific expressed genes (RSEGs) and implied that the anatomical predecessor of the rumen is the esophagus by the most similar expression profile [5], [7]. There is a pressing need to understand how RSCNEs lead to changes in the expression of RSEGs.

One major bottleneck is that the cellular context, target genes (TGs), and mode of gene regulation of RSCNEs are largely unknown. First, the regulatory role of RSCNEs could be spatiotemporally dynamic and highly context-specific. Second, some RSCNEs were located distant (*e.g*., more than 500 kb) from any gene and therefore could not be associated with any TGs using standard the closest transcription start site (TSS) approaches, such as GREAT [8]. This problem is emphasized by a recent finding that a non-coding region associated with a human craniofacial disorder causally affects the expression of *SOX9* at a distance of up to 1.45 Mb during a restricted time window of facial progenitor development [9]. This example motivated us to interpret the function of RSCNEs by uncovering gene regulatory networks (GRNs) with distal regulations from multi-omics data integration at different developmental time points and in different tissue types.

To tackle the aforementioned challenges, we generated time series of paired gene expression and chromatin accessibility data during rumen and esophagus development in sheep to reconstruct a developmental GRN. Our previous efforts showed that jointly modeling multi-omics data allows us to infer high-quality tissue-specific regulatory networks [10], which can be used to identify key transcription factors (TFs) during differentiation [11], reveal causal regulations [12], and interpret functionally important genetic variants [13]. Taken together, we aim to integrate multi-omics data to reconstruct a genome-wide GRN during different stages of development in an apomorphic organ. Specifically, this allows us to understand how TFs bind to functional RSCNEs to coordinate cell type-specific gene expression of RSEGs and hence to gain further insights into the evolutionary development of new traits.

## Results

### The landscapes of accessible chromatin regions and gene expression during rumen development

**Figure 1:**
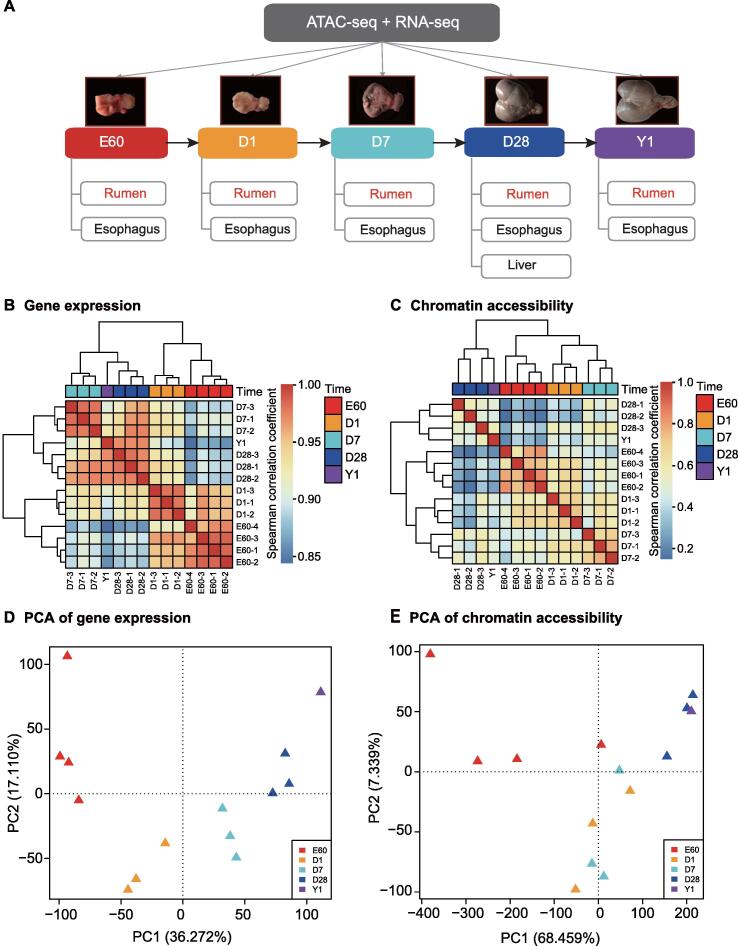
**Time-series dataof paired expression and chromatin accessibilityreveal the regulatory landscape for rumen development** **A.** Experimental design diagram for multi-replicate, multi-tissue, and multi-level omics data profiling during sheep development from E60 to postnatal stages (D1, D7, and D28) to Y1. Hierarchical clustering of gene expression for 14,637 genes (**B**) and chromatin accessibility for 178,651 OCRs (**C**). Unsupervised PCA of rumen gene expression (**D**) and chromatin accessibility (**E**). ATAC-seq, Assay for Transposase-Accessible Chromatin with high-throughput sequencing; RNA-seq, RNA sequencing; E60, embryonic day 60; D1, postnatal day 1; D7, postnatal day 7; D28, postnatal day 28; Y1, adult 1 year; OCR, open chromatin region; PCA, principal component analysis; PC, principal component.

**Supplementary Figure S1:**
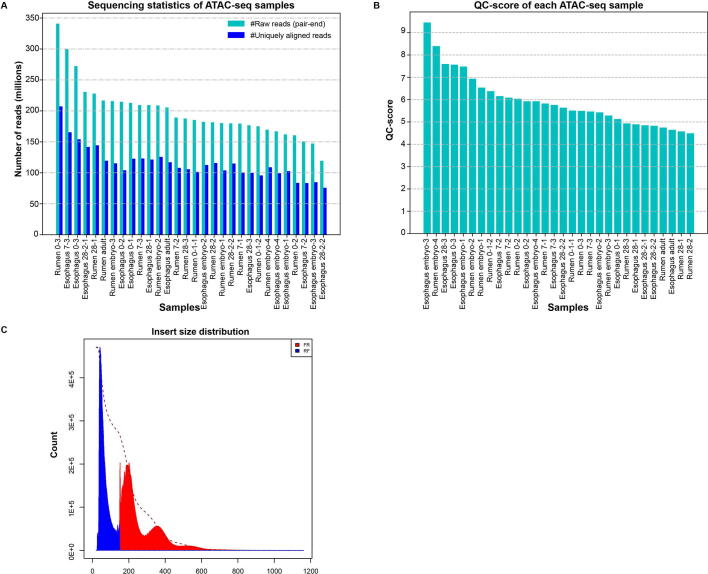
Data quality check for the ATAC-seq samples by their sequence depth, fragment distribution, and QC score A. The number of raw reads and percentile of uniquely aligned reads. The number of raw reads ranges from 110 to 330 million for each ATAC-seq sample, and the percentiles of uniquely aligned reads across all samples is 59% on average. B. QC scores of all ATAC-seq samples. The QC score is defined as the ratio of the total read count at the TSS centered up- and downstream 2 Kbp windows to the randomly selected background [-3 K, -2 K] among all genes. Higher QC score indicates that ATAC-seq signals are enriched in open chromatin regions such as promoters. All the samples were required to reach the standard score of 4, *i.e.*, the fold change was larger than 1. C. Insert size distribution of one example esophagus-0-1. The other 29 samples show a similar pattern. This fragment length distribution reveals a sharp peak at less than 100 bp regions for nucleosome-free fragments, and the second largest peak is within 200 bp for the mono nucleosome fragment. Again, this indicates good data quality. ATAC-seq, Assay for Transposase-Accessible Chromatin with high-throughput sequencing; QC, Quality Control; TSS, Transcription Start Site.

We resolved high-resolution chromatin accessibility and gene expression landscapes during rumen development by collecting ruminal epithelial cells, esophageal epithelial cells, and hepatocyte cells at five stages [embryonic day 60 (E60), postnatal day 1 (D1), postnatal day 7 (D7), postnatal day 28 (D28), and adult 1 year (Y1)] from 14 sheep (Figure 1A). Our experimental design covers the major stages of ruminal epithelium differentiation and development [14], [15] and ensures an exact matching of tissues used for RNA sequencing (RNA-seq) and Assay for Transposase-Accessible Chromatin with high-throughput sequencing (ATAC-seq) libraries. In total, 37 ATAC-seq and 34 RNA-seq datasets, including biological and technical replicates, showed high quality (see Materials and methods; Tables S1 and S2). The ATAC-seq samples have an average of 115 Mb post-quality control (QC) uniquely mapped fragments to the sheep Oar_4.0 genome (Table S1; Figure S1A), which are highly enriched at TSSs (Figure S1B) and show a nucleosome structure consistent distribution (Figure S1C). We obtained 178,651 open chromatin regions (OCRs) across all samples (in average 46,872 peaks per sample) (Table S1).

Hierarchical clustering of gene expression and chromatin accessibility showed that rumen development is a multi-stage biological process (Figure 1B and C). Stages E60 and D1 clustered in one group, and D7, D28, and Y1 clustered in another group by gene expression. Chromatin accessibility patterns further distinguished stages E60 and D1. Principal component analysis (PCA) for 14,637 expressed genes and 178,651 OCRs corroborated this multi-stage pattern (Figure 1D and E). Early developmental stages E60 and D1 showed larger replicate variation than D7, D28, and Y1 at both chromatin accessibility and gene expression levels (Figure 1D and E). In addition, chromatin accessibility showed a smoother trajectory than gene expression during rumen development (Figure 1C).

**Supplementary Figure S2:**
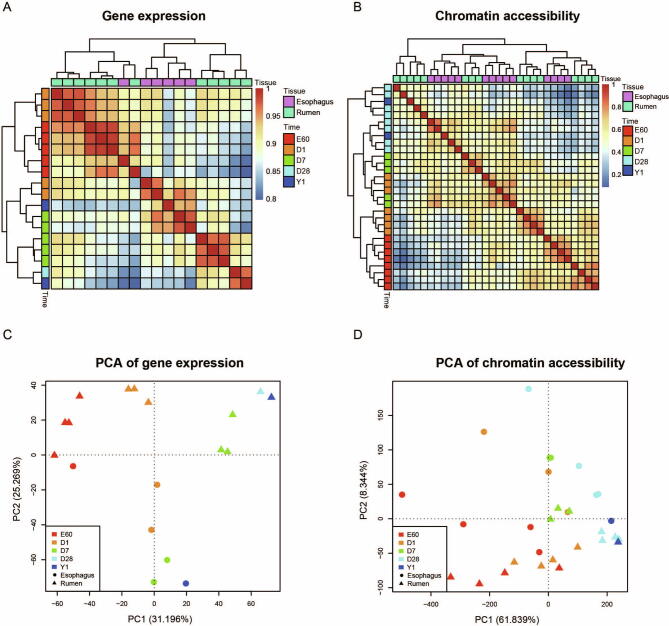
Paired expression and chromatin accessibility time series data reveal the regulatory landscape for rumen and esophagus development. Hierarchical clustering of gene expression (A) and chromatin accessibility (B) of all samples, including biological and technical replicates. PCAof 14,637 genes (C) and 178,651 open chromatin regions (D). The rumen and esophagus show consistent developmental patterns at both gene expression and chromatin accessibility levels. The variation between the rumen and esophagus is less than the variation among developmental stages. PCA, Principal component analysis.

The esophagus showed very similar multi-stage development (Figure S2A and B). PCA indicated a larger variance in developmental stages (PC1 32%) and a smaller variance among tissue types (PC2 25%) (Figure S2C and D). This pattern is consistent with a previous study showing that gene expression divergence between tissues/cell types increases as development progresses [16]. Importantly, our chromatin accessibility data mirror this pattern, *i.e.*, the similarity in chromatin accessibility distribution between the two tissues declines as development progresses.

### Active-RSCNEs serve as enhancers in the process of rumen development and evolution

**Figure 2:**
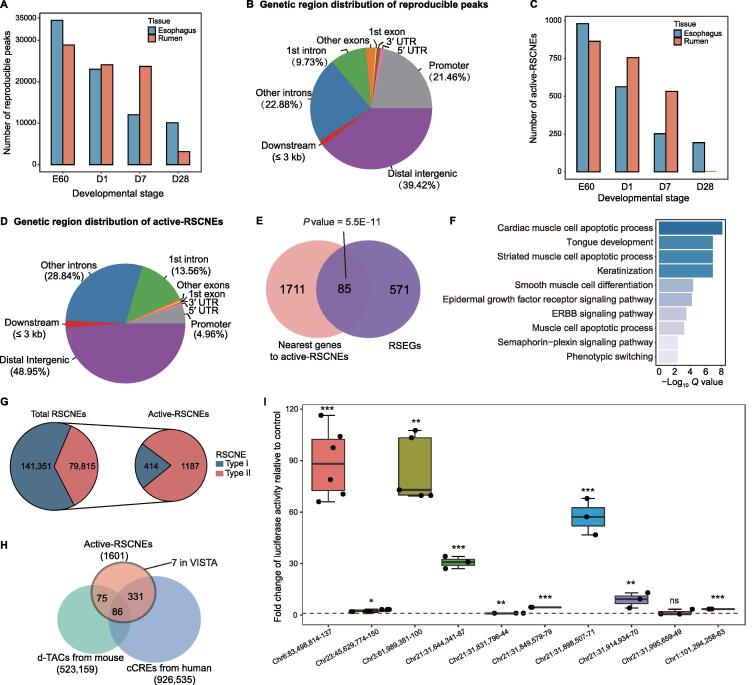
**Characterization of active-RSCNEs as developmental enhancers** **A.** Number of reproducible peaks at each developmental stage in the rumen and esophagus. **B.** Annotating reproducible peaks by location in different genomic regions. **C.** Number of active-RSCNEs at each developmental stage in the rumen and esophagus. **D.** Annotating active-RSCNEs by location in different genomic regions. **E.** The genes nearest to active-RSCNEs are enriched in RSEGs. *P* value is calculated by Fisher’s exact test. **F.** GO enrichment analysis for genes near the active-RSCNEs. **G.** Number of type I and type II RSCNEs in total RSCNEs and active-RSCNEs. **H.** The intersections among active-RSCNEs with enhancers from d-TACs, cCREs, and VISTA. **I.** Luciferase activity assay of 10 active-RSCNEs randomly chosen from 1601 active-RSCNEs. RSCNE, ruminant-specific conserved non-coding element; RSEG, rumen-specific expressed gene; GO, Gene Ontology; UTR, untranslated region; d-TAC, developmental region of transposase-accessible chromatin; cCRE, candidate *cis*-regulatory element.

**Supplementary Figure S3:**
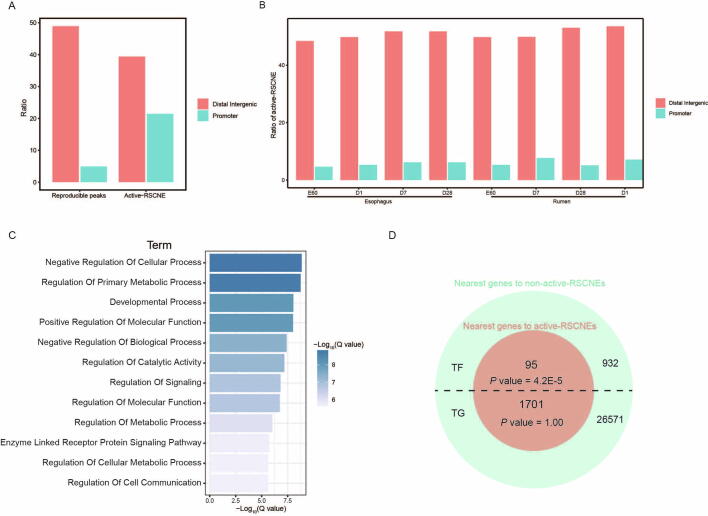
Further characterization of active-RSCNEs A. The percentage of location in distal intergenic and promoter for reproducible peaks and active-RSCNEs. B. The percentage of location in distal intergenic regions and promoters for active-RSCNEs at each developmental stage of the rumen and esophagus. C. GO enrichment analysis of 1796 genes near active-RSCNEs. D. Fisher’s exact test shows that 1796 genes near active-RSCNEs are enriched in TFs (*P* value = 4.20E−5) but not non-TF genes (*P* value = 1.00, no significance). TF, Transcription Factor; TG, Target Gene; GO, Gene Ontology.

We obtained 159,837 reproducible OCRs by intersecting peaks from three replicates for the rumen and esophagus at four developmental stages. The number of reproducible OCRs was the largest at stage E60 (approximately 40%) and decreased along the developmental stages (Figure 2A), which is consistent with the observation of higher amounts of accessible chromatin at the embryonic stage [17]. Most reproducible OCRs were located at distal intergenic (39.42%), intron (32.61%), and promoter (±3 kb from TSS; 21.46%) regions (Figure 2B). After overlapping the OCRs with 221,166 RSCNEs from ruminant comparative genomics analysis [5], we identified 1601 active-RSCNEs with an average length of 82 bp (Table S3). Again, the number of active-RSCNEs decreased during the developmental stages in both the rumen and esophagus (Figure 2C). They were mainly located in distal intergenic (48.95%), intron (42.40%), and promoter (4.96%) regions (Figure 2D). Compared with all reproducible OCRs, active-RSCNEs were less abundant in promoter regions by 15% (Figure S3A), and the esophagus showed a consistent trend (Figure S3B). This suggests that active-RSCNEs tend to function as distal elements during development. In addition, our observation that most active-RSCNEs are found in early developmental stages (> 90% in E60, D1, and D7) emphasizes the importance of early developmental cellular context for interpreting the regulatory role of conserved non-coding elements (CNEs).

We next associated the 1601 active-RSCNEs with their 1796 genes nearby. Gene Ontology (GO) analysis of these genes showed enrichment in terms such as “primary metabolic process”, “catalytic activity”, and “regulation of signaling” (Figure S3C). Moreover, TFs were significantly enriched in these 1796 genes (Figure S3D; Fisher’s exact test, *P* = 4.20E−4). These 1796 genes overlapped with 656 RSEGs by 85 genes (Figure 2E; Fisher’s exact test, *P* value = 5.50E−11) that were enriched in “cardiac muscle cell apoptotic process”, “tongue development”, and “keratinization” (Figure 2F).

The 1601 active-RSCNEs are composed of 414 type I and 1187 type II RSCNEs (Figure 2G; Table S3). Type I RSCNEs have no known orthologs in non-ruminant outgroups, and type II orthologs exhibit significantly higher substitution rates among outgroups [5]. The ratio between type I and type II active-RSCNEs is ∼ 0.35, which is 4-fold lower than that of all RSCNEs (a type I/type II ratio of ∼ 1.77) (Figure 2G). This surprising fact suggests that type II RSCNEs tend to be more activated in the developmental stage than type I RSCNEs. Because of the deeper evolutionary origin of type II RSCNEs, they are more likely to function by altering existing regulatory elements. Furthermore, we found that active-RSCNEs are enriched for binding motifs of transcriptional regulators known to play a vital role in rumen development (AP-1, PITX1, TP63, KLF, GRHL, TEAD, OTX, and HOX; 128 motifs with Benjamini *Q* value < 1E−3 are listed in Table S4), suggesting that some active-RSCNEs may act as rumen developmental enhancers.

To assess whether the RSCNEs are likely to play an enhancer role, we next compared our 1601 active-RSCNEs with the 523,159 developmental regions of transposase-accessible chromatin (d-TACs) from mice [18] and 926,535 human enhancers from Encyclopedia of DNA Elements (ENCODE) phase III [19]. Approximately 24% of the active-RSCNEs can be found in these datasets (Figure 2H), and 11 active-RSCNEs show *in vivo* reporter activity according to the VISTA database [20] (Figure 2H). To validate the potential regulatory activity, 10 active-RSCNEs of length ∼ 300 bp were randomly selected and assessed for enhancer activity detection in both sheep and goat fibroblasts *in vitro*. Nine of them showed significantly higher luciferase transcriptional activation than the pGL3-promoter control (*t*-test, *P* < 0.05; Figure 2I). Collectively, these results suggest that active-RSCNEs potentially serve as enhancers in the process of rumen development and evolution.

### CNE interpretation method by GRN

**Figure 3:**
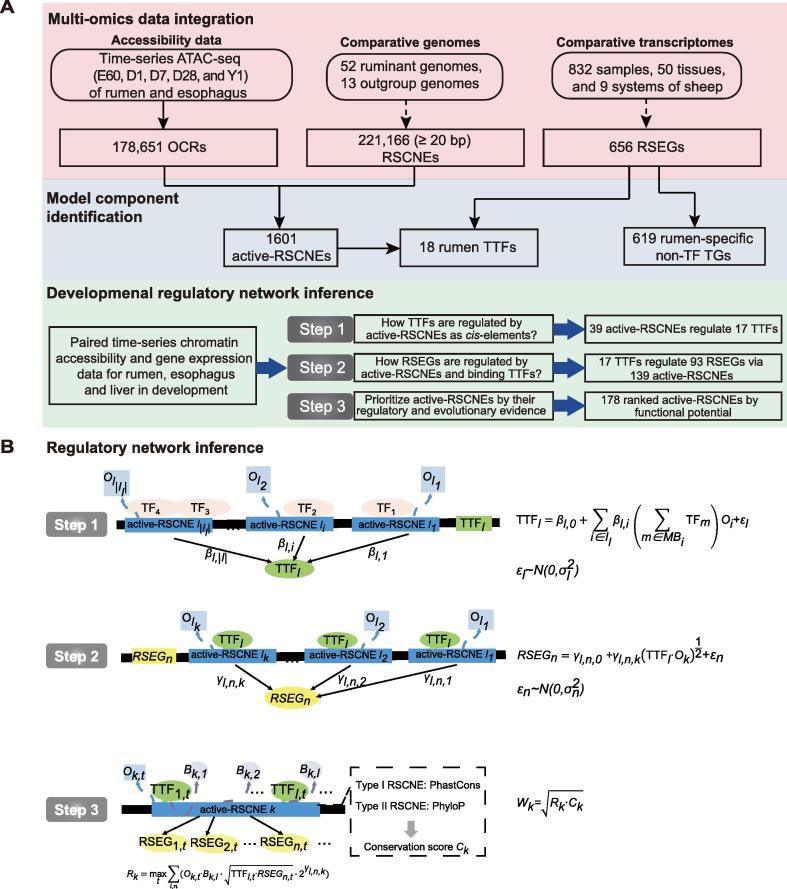
**CNEReg interprets RSCNEs by reconstructing the developmental regulatory network** **A.** CNEReg inputs paired time-series gene expression and chromatin accessibility data, ruminant comparative genomes, and comparative transcriptomes, and outputs the developmental regulatory network of active-RSCNEs. Three major steps of CNEReg include multi-omics data integration, model component identification, and developmental regulatory network inference. **B.** The developmental regulatory network reconstruction is further illustrated in three steps. Step 1: inferring the upstream regulation of rumen TTFs. Step 2: inferring the downstream regulation of TTFs to TGs via active-RSCNEs. Step 3: deriving active-RSCNE’s functional influence score by integrating regulatory strength in the network and evolutionary conservation score. The model components and notations of CNEReg are detailed in Table 1. TG, target gene; TF, transcription factor; TTF, toolkit transcription factor; CNEReg, conserved non-coding element interpretation method to integrate multi-omics data into gene regulatory network; RSCNE, ruminant-specific conserved non-coding element.

After finding that active-RSCNEs may function as enhancers and hence have significant impacts on morphological evolution [21], we next developed the CNE interpretation method to integrate multi-omics data into gene regulatory network (CNEReg) as an evolutionarily conserved non-coding element interpretation method. The method works by modeling the paired gene expression and chromatin accessibility data during rumen and esophagus development and consolidating them into a GRN. A GRN helps to understand in detail the process of TF binding to active-RSCNEs and how this leads to the cell type-specific activation of RSEGs during different stages of development. CNEReg takes as input a set of paired time-series gene expression and chromatin accessibility data, ruminant comparative genomes, and comparative transcriptomes and outputs the developmental regulatory network of the active-RSCNEs. The three major steps of CNEReg include multi-omics data integration, model component identification, and developmental regulatory network inference (Figure 3A and B; see Materials and methods). The major steps and results of developmental regulatory network reconstruction are illustrated in the following sections.

### Identifying TTFs during rumen development and evolution

**Figure 4:**
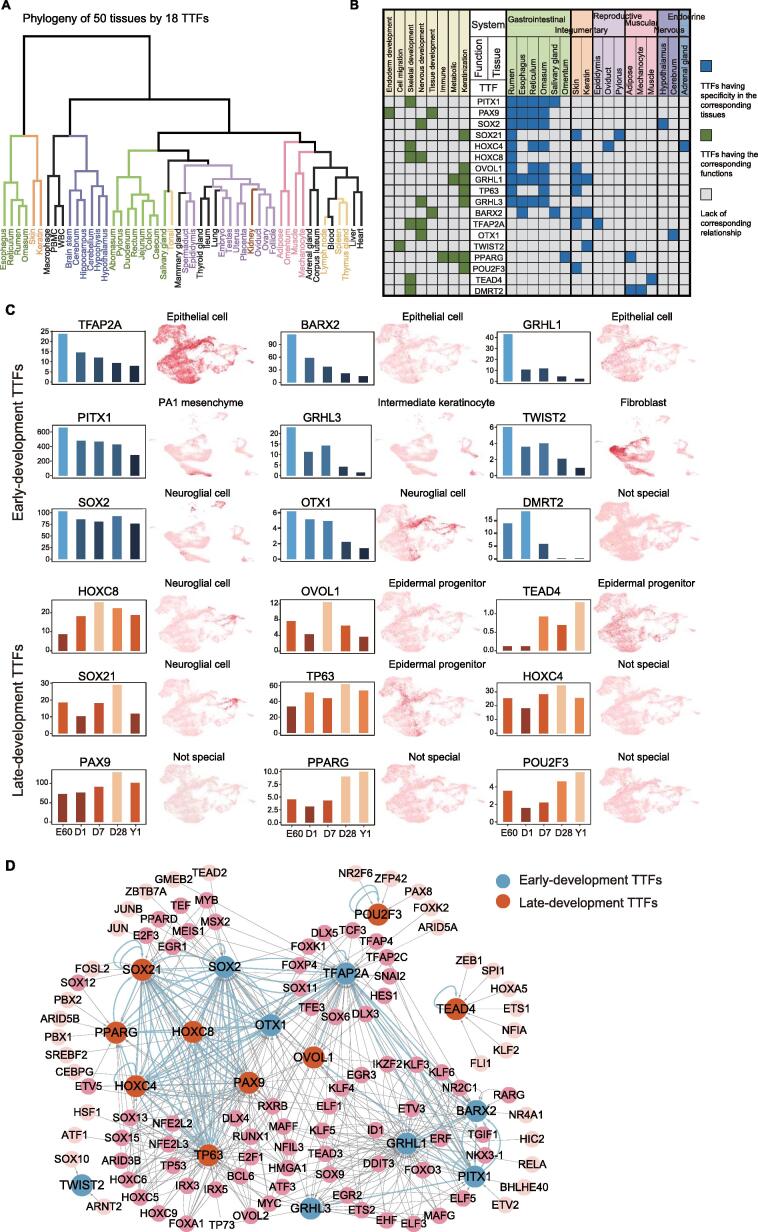
**18 rumen TTFs and their upstream regulations** **A.** Phylogeny of 50 tissues from sheep based on the expression of 18 rumen TTFs groups the samples well by different lineages and biological systems. **B.** Biological functions of 18 rumen TTFs (marked in green) and the tissue with high expression (marked in blue). Tissues are grouped and colored by their lineages. **C.** 18 rumen TTFs are grouped into early-development (cold-colored) and late-development (warm-colored) by their dynamic expression patterns during the developmental stages. In addition, 18 rumen TTFs associated with specific cell type names were visualized by a uniform manifold approximation and projection plot in skin organoid scRNA-seq data. **D.** The upstream gene regulatory network of rumen TTFs shows the candidate TFs with statistical significance. Nodes are colored by early- and late-development TTFs. Blue edges highlight the regulatory relationship among TTFs.

We proposed toolkit transcription factors (TTFs) as the core concept of CNEReg and developed a computational pipeline to define and discover the developmental genetic TTFs in evo-devo that may control development, pattern formulation and identity of body parts, and recruit novel function (details in Materials and methods). We first separated 37 TFs from 619 non-TF TGs in 656 RSEGs. These 37 TFs were further filtered by a more stringent expression specificity Jensen–Shannon Median expression Score (JMS) and were required to have nearby active-RSCNEs 1 Mb upstream or downstream of the TSS (see Materials and methods). Finally, 18 TTFs were defined (Table S5). Their expression profile phylogeny well recovered the tissue lineage system (Figure 4A). Rumen was clustered the closest to the reticulum, omasum, esophagus, and then skin and other keratin tissues, which is consistent with the basic stratified epithelium shared in the rumen with skin. These 18 TTFs also well represented the major functions of the rumen associated with other tissue systems, including the gastrointestinal system, integumentary system, reproductive system, muscular system, nervous system, and endocrine system (Figure 4B).

We observed that the rumen recruited TTFs from multiple tissues to drive gene expression. More TTFs were expressed from the gastrointestinal system than from other systems. For example, paired box protein 9 (PAX9) is a known key TF during esophagus differentiation that may play an important role in the origin of rumen from the esophagus [22]. The homeobox family TFs HOXC8 and HOXC4, together with PITX1, are key developmental regulators of specific positional identities on the anterior-posterior axis [23], [24]. The other four TTFs, OVOL1, SOX21, TFAP2A, and TP63, are from the integumentary system and serve as master regulators in the regulation of epithelial development and differentiation [25], [26], [27], [28].

We classified 18 TTFs into two types according to their dynamic gene expression pattern during rumen development. PITX1, BARX2, SOX2, GRHL1, GRHL3, TFAP2A, OTX1, DMRT2, and TWIST2 are early-development TTFs showing the highest expression at E60 or D1 (Figure 4C). In contrast, PAX9, TP63, HOXC4, SOX21, HOXC8, OVOL1, PPARG, POU2F3, and TEAD4 were late-development TTFs and were highly expressed at D7, D28, or Y1 (Figure 4C). We further associated those TTFs with 6 cell types by scRNA-seq data in the skin organoid culture system [29], and they showed specific expression levels at single-cell resolution in a complex skin organ model by reprogramming pluripotent stem cells. For example, TWIST2 is specifically expressed in fibroblast (Figure 4C). TWIST2 remodels chromatin accessibility to regulate the maturation of fibroblasts [30] and is required for epithelial–mesenchymal transition [31]. Its high expression at the early developmental stage of the rumen may relate to the ruminal epithelial development. Totally, our results indicated these identified TTFs act important regulatory roles in diverse cell types of the rumen.

### Constructing upstream and downstream regulations of rumen TTFs

To explore how TTFs are regulated and recruited, we scanned the active-RSCNEs near TTFs for sequence-specific TF motif binding by HOMER [32], retained those TFs correlating well with TTFs (Spearman’s correlation coefficient > 0.6 across RNA-seq samples), and fitted a linear regression model integrating our paired expression and chromatin accessibility data to reveal upstream regulators of 18 rumen TTFs (Figure 3B; see Materials and methods). The resulting upstream regulatory network of TTFs (Figure 4D) identified 39 active-RSCNEs (15 type I and 24 type II) bound by 113 TFs for 18 TTFs (Table S6). GRHL1, an important regulator of keratin expression [33], is regulated by 31 TFs via 6 active-RSCNEs, suggesting its potential roles in rumen development.

**Figure 5:**
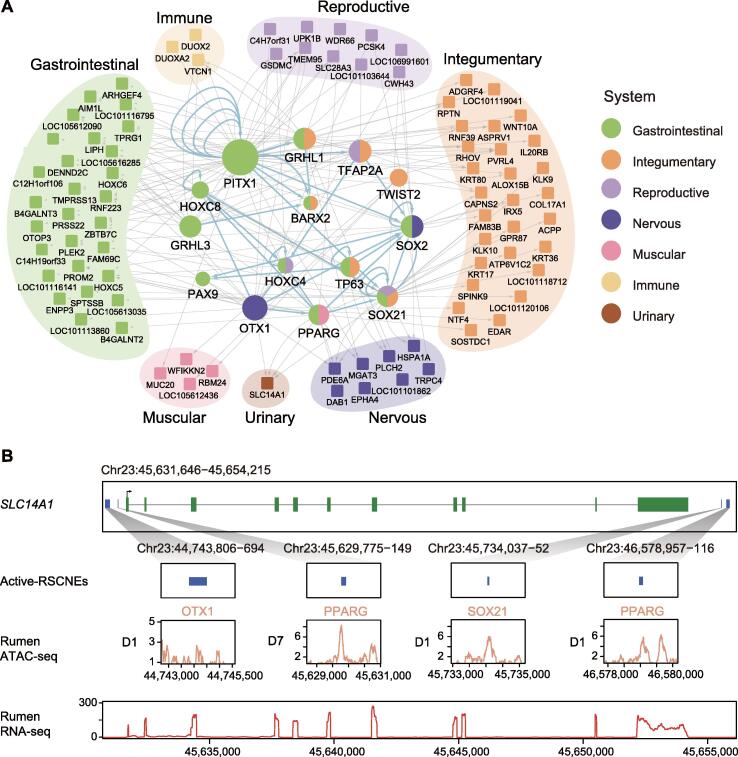
**Downstream regulatory network of rumen TTFs** **A.** Downstream regulatory network with 14 rumen TTFs regulating 93 TGs via 139 active-RSCNEs. TTFs are colored by the tissue they are highly expressed, and TGs are annotated and colored by their biological system. **B.** An example from the regulatory network shows that *SLC14A1* is regulated by four active-RSCNEs with TTF motifs. The expression and chromatin accessibility tracks are derived from rumen ATAC-seq (D1 or D7) and RNA-seq data (Y1).

To explore the regulatory roles of these 18 TTFs, we first scanned 1440 active-RSCNEs located 1 Mb upstream or downstream around 512 RSEGs [fragments per kilobase per million mapped reads (FPKM) > 1 in at least one development stage] by HOMER [32] for binding sites of the 18 rumen TTFs. Then, a linear regression model quantitatively associated the accessibility of active-RSCNEs with the expression of TTFs and RSEGs (Figure 3B; see Materials and methods). The resulting downstream regulatory network of TTFs linked 139 active-RSCNEs (26 type I and 113 type II) with 14 TTFs and 93 RSEGs (Figure 5A; Table S7). RSEGs were categorized into seven different tissue systems by their expression specificity [5], [7]. The gastrointestinal and integumentary systems both have 28 RSEGs that are functionally enriched in the hair/molting cycle process (Fisher’s exact test, adjusted *P* = 1.50E−2) and regulation of antimicrobial peptide production (Fisher’s exact test, adjusted *P* = 3.58E−6). This is consistent with our previous finding that the rumen evolved several important antibacterial functions specifically managing the microbiome composition [2]. The *SLC14A1* gene was specifically highly expressed in the rumen and hypothesized to be recruited from the urinary system (Figure 5A). CNEReg identified four active-RSCNEs bound by three TTFs, OTX1, PPARG, and SOX21, to regulate *SLC14A1* (Figure 5B).

**Supplementary Figure S4:**
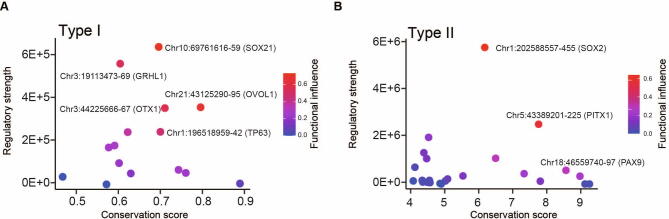
Relationships between the regulatory strength and the conservation score of the TTF upstream network Scatter diagrams show the regulatory strength (y-axis), conservation score (x-axis), and functional influence score (node color) of type I (A) and type II (B) active-RSCNEs with regulated genes in the network shown in Figure 4.

**Supplementary Figure S5:**
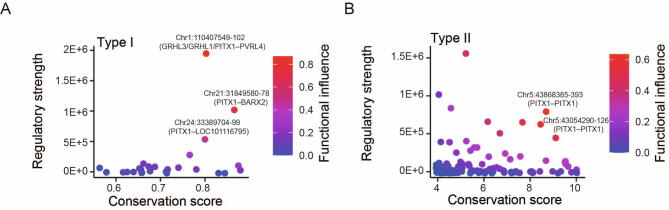
Relationships between the regulatory strength and the conservation score of the TTF downstream network Scatter diagrams show the regulatory strength (y-axis), conservation score (x-axis), and functional influence score (node color) of type I (A) and type II (B) active-RSCNEs with regulated genes from the network in Figure 5.

**Supplementary Figure S6:**
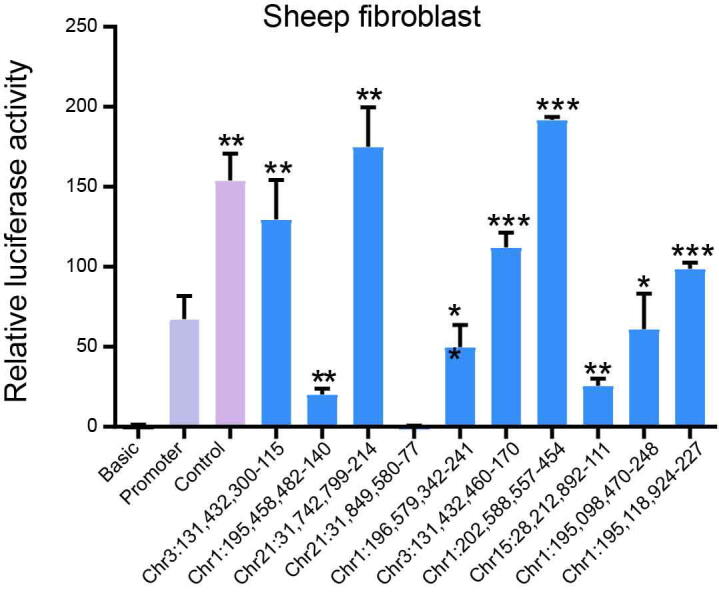
Luciferase activity assays of 10 active-RSCNEs with the top functional influence score Nine of 10 showed significant regulatory activity in the PGL-3 promoter (one-sided *t*-test, *, *P* < 0.05, **, *P* < 0.01, ***, *P* < 0.001).

CNEReg designed a functional influence score by integrating regulation and conservation in evolution (Figure 3B; see Materials and methods) and ranked the active-RSCNEs in TTF upstream (Figure S4; Table S6) and downstream networks (Figure S5; Table S7). Then, we selected the top 10 active-RSCNEs for enhancer activity detection in sheep fibroblasts *in vitro*. Nine of ten showed significantly higher luciferase transcriptional activation than the pGL3-promoter control (*t*-test, *P* < 0.05) (Figure S6). Collectively, CNEReg provides a high-quality developmental regulatory network to study rumen evolution.

### Regulatory sub-network underlying the rumen and esophagus divergence

**Figure 6:**
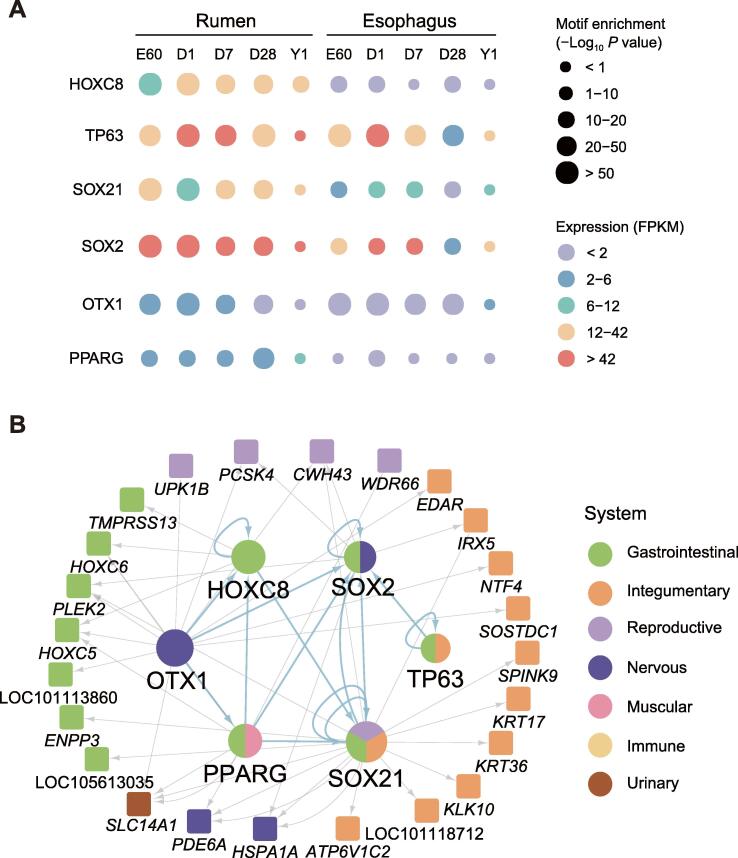
**Regulatory network sheds light on the difference between the rumen and esophagus in the development** **A.** Dynamics across stages for the six differential TTFs between the rumen and esophagus by integrating motif enrichment in differential ATAC-seq peaks and gene expression levels. **B.** Downstream regulatory sub-network of six differential rumen TTFs. FPKM, fragments per kilobase of exon per million mapped fragments.

**Supplementary Figure S7:**
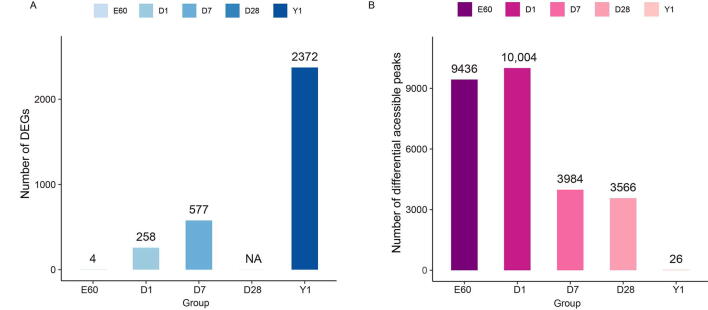
Differentially expressed genes and differentially accessible peaks between the rumen and esophagus at each stage A. Barplots showing the number of differentially expressed genes between the rumen and esophagus at each stage. B. Barplots showing the number of differentially accessible peaks between the rumen and esophagus at each stage. DEG, Differentially Expressed Gene.

We previously hypothesized that the anatomical predecessor of the rumen is the esophagus based on their similar expression profile compared with 49 other tissues [5], [7]. It is therefore of interest to identify the gene regulatory network underlying the differentiation between the rumen and esophagus. We first identified differentially expressed genes (4, 258, 577, and 2372 for E60, D1, D7, and Y1, respectively, in Figure S7A) and differentially accessible regions (9436, 10,004, 3984, 3566, and 26 for E60, D1, D7, D28, and Y1, respectively, in Figure S7B) between the rumen and esophagus at each developmental stage. Then, we identified six TTFs (PPARG, SOX21, TP63, OTX1, SOX2, and HOXC8) showing both significant differences in expression and in motifs enriched within the rumen OCRs (Figure 6A; see Materials and methods). HOXC8 showed the largest difference at the earliest developmental stage, both in expression level and motif enrichment, and SOX21, SOX2, OTX1, and PPARG showed similar trends. TP63 differentiates from D7, in which the gene expression level and motif enrichment decline quickly in the esophagus but not in the rumen.

We extracted the six differential TTFs from the TTF downstream regulatory network to form a regulatory sub-network that also included 24 differentially expressed RSEGs and 38 active-RSCNEs (Figure 6B; Table S8). The 24 differentially expressed RSEGs were classified into gastrointestinal, integumentary, reproductive, nervous, muscular, immune, and urinary systems, and 10 of 24 non-TF RSEGs were classified into integumentary systems. Seven non-TF RSEGs (*KRT17*, *KRT36*, LOC101118712, *ATP6V1C2*, *KLK10*, *SPINK9*, and *IRX*) were regulated by SOX21. A previous study revealed that SOX21 could determine the fate of ectodermal organs and control epithelial differentiation [28]. We observed that SOX21 binds to RSCNE with genomic coordinates “chr11:40325877-150” to regulate the expression of KRT17, KRT36, and LOC101118712. The functional influence of RSCNE with genomic coordinates “chr11:40325877-150” was ranked at the top of all type II active-RSCNEs in the differentially regulatory sub-network (Table S8). These RSEGs were enriched in epidermal development, formation of anatomical boundaries, and urea transmembrane transport biological processes (Table S9), which are consistent with the functional differences between the rumen and esophagus. The 38 active-RSCNEs may imply the potential genetic basis of rumen origin and evolution from the esophagus.

### Transposable elements may rewire the GRN through active-RSCNEs

After interpreting active-RSCNEs as important regulators of TTFs and RSEGs in rumen development, we next addressed the genomic origin of the active-RSCNEs. Transposable elements (TEs) are known to constitute a high proportion of taxonomy-specific CNEs, play a central role in rewiring gene regulatory networks, and facilitate the novel or rapid evolution of ecologically relevant traits [34], [35]. Hence, we estimated the percentage of active-RSCNEs may be derived from TEs. Among 39 and 139 active-RSCNEs in the TTF upstream and downstream networks, we identified 6 (15.38%) and 12 (8.6%) TEs, respectively. This gives a 1.8-fold enrichment of TEs in active-RSCNEs associated with TTFs relative to non-TTF RSEGs. At the gene level, 6 of 18 TTFs (33.33%) and 12 of 93 RSEGs (12.90%) are regulated by TEs via active-RSCNEs. This gives a 2.58-fold enrichment of TEs associated with TTFs relative to RSEGs. If we associated the TEs with RSEGs by their proximity in genome coordinates, there were 85 TEs around all 656 RSEGs (±200 kb), *i.e.*, 13% of RSEGs are associated with TEs in average. However, 33.3% TTFs are associated with TEs, and this is 1.56-fold higher than RSEGs. Together, our data suggest that TEs may recruit TTFs and rewire the regulatory network to give rise to trait novelties.

## Discussion

The evolution of new traits is driven by several types of genetic reprogramming, including mutations in protein-coding genes and post-transcriptional mechanisms, the transformation of regulatory elements, such as promoters and enhancers, and the recruitment of gene expression from other organs [36], [37]. Mutations in non-coding regulatory regions are believed to selectively perturb TG expression in a specific tissue context and thereby circumvent any pleiotropic effects from protein-coding mutations [38]. Recent advances in comparative genomics, along with the increased availability of whole genome sequences, have led to the identification of many CNEs, which are assumed to have regulatory functions [1], [6], [39]. Therefore, the time is ripe for an analytical framework to investigate the regulatory role of such CNEs.

Biologically, we propose a model of gene expression recruitment by CNEs. Our results show how CNEs can regulate gene expression as either *trans*-regulatory elements (TTFs in this study) or *cis*-regulatory elements (CREs; active-RSCNEs in this study) of TGs (RSEGs). Methodologically, CNEReg provides a framework to integrate comparative genomics, comparative transcriptomic, and multi-omics data to interpret CNEs by GRN. On the one hand, GRN identifies TTFs and active-RSCNEs as hypotheses, which need to be pursued by *in vitro* and *in vivo* functional studies. On the other hand, GRN presents the global picture of how the rumen recruits gene expression from other tissues by activating RSCNEs to achieve many traits. This allows us to explore many biological hypotheses and rank candidates for further functional study. As an example, we reconstructed a sub-network underlying rumen and esophagus divergence, which could further interpret the differences between the rumen and its ancestral organ. The identified TTFs and active-RSCNEs in the sub-network may account for the origin and evolution of the rumen. For example, as one of the genes in Hox gene family, *HOXC8* has been implicated in the divergence of axial morphology [40]. The proper expression of Hox genes is essential for the precise patterning of the body plan. *HOXC8* shows differences in gene expression and motif enrichment between the rumen and esophagus during development. These results indicate that the rumen employed a different gene regulatory program when differentiating from the esophagus.

Our method for systematically interpreting conserved *cis*-regulatory sequences in non-coding regions by integrating developmental multi-omics data will have a broad interest in other applications. For example, the Zoonomia project describes a whole-genome alignment of 240 species comprising representatives from more than 80% of mammalian families [41]. The bird 10,000 Genomes Project provides a comparative genome dataset for 363 genomes from 92.4% of bird families [42]. Recently, 6.9 million CNEs from many vertebrate genomes have been collected into the dbCNS and await interpretation [43].

Our work is limited in several aspects. CNEReg infers gene regulation as the interaction of TFs with accessible DNA regions in development and relies on the correlation of gene expression and chromatin accessibility across samples. A much deeper understanding can be revealed by ChIP-seq data and 3D chromatin interaction data to provide physical enhancer-promoter interactions. In addition, time course regulatory analysis of omics data measured at shorter and closer developmental stages will help us to infer more accurate regulatory network [12]. Furthermore, developmental samples are known as a heterogeneous mixture of many cell types, and it will be fruitful to infer the GRNs of the underlying cell types based on scATAC-seq and scRNA-seq data [10].

In conclusion, CNEReg is demonstrated as a systematic approach to understanding the large-scale maps of CNEs by modeling omics data over development for its act on gene regulation. We see the potential that CNEReg can be generalized to understand the complex traits or the origin and evolution of vertebrate organs with multi-omics data generated in proper time and space. Our method allows evo-devo thinking in how gene regulation could evolve and shape animal evolution.

## Materials and methods

### CNEReg infers developmental regulatory network to interpret CNEs

CNEReg aims to systematically fill the gap between CNEs and their significantly impacted morphology in evolution. This is done by reconstructing a developmental regulatory network by paired time series of paired gene expression and chromatin accessibility data. Particularly in sheep, CNEs are RSCNEs, and morphology is the innovation of the rumen organ, which is further denoted by the set of rumen-specific genes. We reconstructed a gene regulatory network during rumen development to systematically understand how TFs regulate genes via batteries of RSCNEs, which over development led to the cell type-specific activation of RSEGs.

The main idea of CNEReg is to define those TTFs as major players in evo-devo and to study how those TFs are regulated by RSCNEs and how they utilize RSCNEs to regulate RSEGs. CNEReg models the expression of TGs conditional on the chromatin accessibility of RSCNEs and the expression of TFs. CNEReg is composed of three steps, as shown in Figure 3, and uses three equations to model (1) the expression of TTFs, (2) the expression of RSEGs, and (3) the functional influence of RSCNEs (Figure 3; Table 1).

**Table 1:** Components and notations of the CNEReg model

**Data and variable**	**Notation**	**Example**
Expression of TTFs	$\mathrm{TT}F_{l,t}$ = expression of the *l*-th TTF on *t*-th time point	$\mathrm{TT}F_{HOXC8}$ = 25.48 on D7 in rumen
Expression of TFs	$TF_{m}$ = expression of the *m-*th TF	$TF_{\mathrm{JUN}}$ = 1035.79 on D7 in rumen
Expression of RSEGs	$\mathrm{RSE}G_{n,t}$ = expression of the *n*-th RSEG on *t*-th time point	$\mathrm{RSE}G_{SLC14A1}$ = 42.34 on D7 in rumen
Accessibility of active-RSCNEs	$O_{k,t}$ = openness of the *k*-th active-RSCNE on *t*-th time point	$O_{\mathrm{Chr}1:196579342-242}$ = 18.83 on D7 in rumen
TFs with motif match in an active-RSCNE	${\mathrm{MB}}_{i,l}$ = the set of TFs with significant motif match in *i*-th active-RSCNE	HOXC8 has motif match at active-RSCNE Chr1:196579342–242
Motif matching strength of TFs on RSCNEs	$B_{i,l}$ = sum of −log *P* value of *l*-th TF’s motif on *i*-th active-RSCNE	$B_{\mathrm{Chr}1:196579342-242}$ = 4.28486

*Note*: CNEReg, conserved non-coding element interpretation method to integrate multi-omics data into gene regulatory network; TTF, toolkit transcription factor; TF, transcription factor; RSEG, rumen-specific expressed gene; RSCNE, ruminant-specific conserved non-coding element.

### Defining and identifying TTFs

We identified TTFs by their nearby evolutionally conserved CREs in the genome, expression patterns across tissues, and expression levels in developmental stages. TTFs should satisfy-four conditions: (1) TFs should be rumen-specifically expressed genes (37 TFs in the 656 RSEGs); (2) there should be active-RSCNEs around TFs (±1 Mb, 35 TFs remain); (3) TFs should be expressed (FPKM > 1) at least one time point during rumen development (30 TFs remain); and (4) these TFs should have additional tissue specificity. TFs were ranked by our tissue specificity JMS (see “Defining tissue specificity score” section)*,* and only the TFs for the top 50 specificities in at least one tissue were selected (18 TFs remain). Finally, 18 TFs were identified as TTFs and are listed in Table S5. These TFs played a leading role in rumen development (Table S5) and served as the main component to construct the rumen developmental regulatory network.

### Modeling expression of TTFs

We modeled how TTFs are regulated from paired gene expression and chromatin accessibility data to reconstruct the upstream regulatory network of TTFs. We established a linear regression model as follows to reveal the upstream regulators of the 18 TTFs (schematic illustration in Figure 3 and mathematical notations in Table 1).

(1) $${\mathrm{TTF}}_{l}=\beta_{l,0}+\sum_{i\in I_{l}}\beta_{l,i}\left(\sum_{m\in {\mathrm{MB}}_{i}}{\mathrm{TF}}_{m}\right)O_{i}+\varepsilon_{l},\;\varepsilon_{l}∼\;N\left(0,\sigma_{l}^{2}\right)$$

where ${\mathrm{TTF}}_{l}$ is the expression of the *l*-th TTF; ${\mathrm{MB}}_{i}$ is the set of TFs with significant motif match in the *i*-th active-RSCNE; and ${\mathrm{TF}}_{m}$ is the expression of the *m*-th candidate TF with a binding motif to regulate the *l*-th TTF. The Spearman correlation coefficient between ${\mathrm{TF}}_{m}$ and ${\mathrm{TTF}}_{l}$ is greater than 0.6 [false discovery rate (FDR) *Q* value < 0.01] to ensure the potential regulatory relationship; $O_{i}$ represents the chromatin accessibility score of the *i*-th active-RSCNE within 2 Mb around the *l*-th TTF. β is the parameter to be estimated. If $\beta_{l,i}$ is statistically significant non-zero in the regression analysis, the *i*-th active-RSCNE and its TFs in ${\mathrm{MB}}_{i}$ will be contained in the upstream regulatory network of the l-th TTF.

### Modeling expression of RSEGs

We modeled how the RSEGs are regulated by TTFs and their active-RSCNEs from paired gene expression and chromatin accessibility data, *i.e*., to reconstruct the downstream network regulated by TTFs. We established the linear regression model as follows (schematic illustration in Figure 3 and mathematical notations in Table 1):

(2) $${\mathrm{RSEG}}_{n}=\gamma_{l,n,0}\;+\gamma_{l,n,k}{\left({\mathrm{TTF}}_{l}∙O_{k}\right)}^{\frac{1}{2}}+\varepsilon_{n},\;\varepsilon_{n}∼\;N\left(0,\sigma_{n}^{2}\right)$$

where ${\mathrm{TTF}}_{l}$ is the expression of the *l*-th TTF; $O_{k}$ represents the chromatin accessibility score of the *k*-th active-RSCNE with binding sites of the l-th TTF; and ${\mathrm{RSEG}}_{n}$ is the expression of the *n*-th RSEG with the *k-*th active-RSCNE within approximately 2 Mb. In practice, we determine the downstream regulation relationship with Spearman correlation that can eliminate the outlier values to simplify the calculation. When the Spearman correlation coefficient $\gamma_{l,n,k}$ between ${\mathrm{RSEG}}_{n}$ and ${\left({\mathrm{TTF}}_{l}\cdot O_{k}\right)}^{\frac{1}{2}}$ is greater than 0.7 (FDR *Q* value < 0.01), the *n*-th RSEG is likely to be regulated by the *l*-th TTF through binding to the *k*-th active-RSCNE. The extracted TTF, active-RSCNEs, and RSEG triplets are formed the TTF’s downstream regulatory network.

### Quantifying the functional influence of active-RSCNEs

We quantified the functional influence of active-RSCNEs, ranked the active-RSCNEs, and selected the top active-RSCNEs as experimental candidates. This task can be done by integrating the RSCNE’s conservation score in comparative genomics analysis with its regulatory potential in our developmental regulatory network.

We first collected conservation scores of active-RSCNEs from a comparative genomics study [5]. RSCNEs were classified into two types by their conservation patterns across species. Type I RSCNEs had no outgroup sequence aligned, and type II RSCNEs had orthologous sequences in one or more outgroups but were only conserved in ruminants. For the k-th active-RSCNE, the conservation score $C_{k}$ was calculated by the PhastCons score (type I) or PhyloP score (type II).

We then estimated the regulatory strength of active-RSCNEs in the upstream and downstream regulatory networks of TTFs. An active-RSCNE played a regulatory role in the regulatory network if four conditions were satisfied: (1) this active-RSCNE should be a chromatin-accessible peak; (2) TTFs should bind to this active-RSCNE; (3) RSEGs regulated by this active-RSCNE with TTF binding should be expressed; and (4) the expression of binding TTFs and the accessibility of this active-RSCNE should be correlated with the expression of regulated RSEGs. By combining these four factors, we defined the regulatory strength $R_{k,t}$ of the k-th active-RSCNE at time point t in the regulatory network as follows:

(3) $$R_{k,t}=\sum_{l,n}\left(O_{k,t}∙B_{k,l}∙\sqrt{{\mathrm{TTF}}_{l,t}∙{\mathrm{RSEG}}_{n,t}}∙2^{\gamma_{l,n,k}}\right)$$

where $O_{k,t}$ is the chromatin accessibility score of the k-th active-RSCNE at time point t in the rumen; $B_{k,l}$ is the motif binding strength of the l-th TTF on the k-th active-RSCNE (computed by HOMER); ${\mathrm{TTF}}_{l,t}$ is the expression of the l-th TTF at time point t in the rumen; ${\mathrm{RSEG}}_{n,t}$ is the expression of the n-th RSEG at time point t in the rumen; and $\gamma_{l,n,k}$ is the Spearman correlation coefficient between ${\mathrm{RSEG}}_{n}$ and ${\left({\mathrm{TTF}}_{l}\cdot O_{k}\right)}^{\frac{1}{2}}$ from the regulatory network. Then, the regulatory strength $R_{k}$ of the k-th active-RSCNE was defined as the maximum value across all time points in rumen samples as follows:

(4) $$R_{k}=\max_{t}R_{k,t}$$

The regulatory strength $R_{k}$ is from the multi-omics data in development, and the conservation score $C_{k}$ is from multi-genome data across species. The two measures are at the regulation level and genome sequence level, respectively. They can be naturally assumed to be independent of each other. In practice, we found that the regulatory strength and the conservation score were quite complementary to each other (Figures S4 and S5) for active-RSCNEs. Hence, we defined the functional influence $W_{k}$ of the k-th active-RSCNE as the geometric mean of the regulatory strength $R_{k}$ and the conservation score $C_{k}$ as follows:

(5) $$W_{k}=\sqrt{R_{k}\cdot C_{k}}$$

This functional influence score allows us to prioritize active-RSCNEs by approximating their importance in rumen innovation.

### Defining tissue specificity score

Specificity illustrates the property that genes are functional in one particular biological context compared with other contexts. For our transcriptomics data across 50 tissues in sheep, genes highly expressed in only one or several tissues but not expressed in other tissues were defined as tissue specific. Our gene expression matrix had 23,126 rows (the number of expressed genes) and 830 columns (the number of samples sequenced in 50 sheep tissues with each tissue having several biological replicates; Table S10).

To quantify the tissue specificity, we proposed a Jensen–Shannon Median expression Score (JMS) for a gene in certain tissues to combine the gene expression level with a Jensen–Shannon divergence (JSD) value as follows:

(6) $$\mathrm{JMS}=\frac{\sqrt[{3}]{\mathrm{med}(G)}}{\mathrm{JSD}}$$

where med(G) represents the gene’s median expression in a certain tissue across biological replicates. $\sqrt[{3}]{\mathrm{med}(G)}$ can guarantee that the numerator and denominator are of the same magnitude. JSD is the Jensen–Shannon divergence to evaluate the gene’s expression specificity introduced in [44]. It adopts an entropy-based measure to assess the similarity between two probability distribution statistics as follows:

(7) $$\mathrm{JSD}(P|\left|Q\right)=\frac{1}{2}\left(\sum_{k=1}^{n}x_{k}log\frac{2x_{k}}{x_{k}+y_{k}}+\sum_{k=1}^{n}y_{k}log\frac{2y_{k}}{x_{k}+y_{k}}\right)$$

where $P=(x_{1},x_{2},\cdots ,x_{n})$ and $Q=(y_{1},y_{2},\cdots ,y_{n})$ are two probability distributions constructed from our gene expression values across tissues. *n* is the number of samples. Given each row of our gene expression matrix, we normalized the gene expression vector, *i.e.*, each element in this vector was divided by the sum of all elements. For a given gene, $Q=(y_{1},y_{2},\cdots ,y_{n})$ is its corresponding normalized row vector. Given the tissue we are interested, $P=(x_{1},x_{2},\cdots ,x_{n})$ is constructed as a control vector whose components are $\frac{1}{m}$ in the given tissue with m replicates and 0 in other tissues. Finally, the JSD will be calculated as the divergence between *P* and *Q* for a certain gene in certain tissue. The smaller the JSD value was, the more specific this gene was in this tissue.

In summary, our JMS provided a relative specificity score by a nonlinear measure of divergence by emphasizing significantly highly expressed genes in certain tissues to enhance specificity. This JMS allows us to better explore the TTF expression patterns and recruitment of genes based on tissue specificity.

### Differential regulatory network construction between rumen and esophagus

We constructed a differential regulatory network between rumen and esophagus by extracting differential RSEGs, differential TTFs, and active-RSCNEs associated sub-network from the regulatory network of TTFs. The differential RSEGs and differential TTFs are defined as follows.

#### Differential RSEGs between rumen and esophagus

We used the R packages “*limma*” and “edgeR” to extract differential genes at four developmental time points (E60/D1/D7/D28) with thresholds of FDR < 0.05 and log_2_ fold change (FC) > 1 (FC of FPKM in the rumen relative to that in the esophagus). It was noted that at time point Y1, we had only one biological replicate for RNA-seq data in the rumen and esophagus separately, and we could not perform an F test on these two samples. Instead, we identified genes with FPKM > 2 in the rumen and FC > 2 as differential genes. Then, we combined differential genes at five time points to obtain differential gene sets between rumen and esophagus. Differential RSEGs between rumen and esophagus were the intersection of the differential gene set and the RSEG set in the regulatory network of TTFs.

#### Differential accessible peaks between rumen and esophagus

We implemented the R packages “*limma*” and “edgeR” to obtain differential accessible peaks between rumen and esophagus at five developmental time points (E60/D1/D7/D28/Y1) with thresholds of FDR < 0.05 and |log_2_ FC| > 1.

#### Differential TTFs between rumen and esophagus

We first collected 1027 TFs of sheep from animalTFDB3.0 (http://bioinfo.life.hust.edu.cn/AnimalTFDB/#!/). The 15,835 expressed genes in the rumen and esophagus were intersected with these 1027 TFs to obtain 768 TFs for the following analysis. We used HOMER to find TFs binding to the differential accessible peaks with threshold of −log_10_ *P* value > 6 at each time point. Then, we used the R packages “*limma*” and “edgeR” to obtain differentially expressed TFs at four time points (E60/D1/D7/D28) with thresholds of FDR < 0.05 and log_2_ FC > 1. We identified differentially expressed TFs at time point Y1 with threshold FPKM > 2 in the rumen and FC > 2. The differential TF set was defined as the intersection of TFs binding to differential accessible peaks and differentially expressed TFs. Differential TTFs between the rumen and esophagus were the intersection of the differential TF set and TTF set in the regulatory network of TTFs.

### Hierarchical clustering and PCA

We performed hierarchical clustering on the gene expression and peak chromatin accessibility profiles in 14 rumen samples at five time points (E60/D1/D7/D28/Y1). Heatmap was plotted by the R package “pheatmap” with “correlation” as the distance measure and “complete” as the clustering method. Then, we performed dimensional reduction by PCA with the R function “prcomp”. The gene expression and chromatin accessibility value were log-transformed as log_2_ (FPKM+1) and log_2_ (openness+1) as input. The openness score was calculated for each peak under each condition as the FC of read number per base pair [10]. The first two principal components are shown in Figure 1D and E.

### Collecting samples for ATAC-seq and RNA-seq

We collected a total of 37 samples of the rumen, esophagus epithelium tissues, and liver tissues from 14 Hu sheep, including five time points (E60/D1/D7/D28/Y1) from XiLaiYuan Ecological Agriculture Co., Ltd. (Taizhou, China). All samples were rinsed with PBS and soaked in cold 1× PBS supplemented with penicillin–streptomycin (Catalog No. 15140122, Gibco, Grand Island, NY). All animals were slaughtered under the guidelines of the Northwest A&F University Animal Care Committee.

### ATAC-seq library preparation, sequencing, and analysis

All the protocols for ATAC-seq used in this study have been described previously [2]. Hence, we described the experimental procedures and approaches here briefly. The ruminal and esophageal epithelial cells were separated manually from the muscular layer. Then, 0.25% trypsin pre-warmed in 37 °C water bath was used to digest the ruminal and esophageal epithelial cells. Dulbecco’s modified eagle medium (DMEM) solution was added to the cell suspension to adjust the cell density to 1 × 10^6^ cells/ml. To prepare nuclei, the cells were lysed using cold lysis buffer (10 mM Tris-HCl pH 7.4, 10 mM NaCl, 3 mM MgCl_2_, and 0.1% NP40). Subsequently, the transposition reaction was conducted using the TruePrep DNA library prep kit v2 for Illumina (Catalog No. TD501-01/02, Vazyme, Nanjing, China). The samples were immediately purified using a Qiagen MinElute kit. PCR was performed to amplify the library for 14 cycles according to the manufacturer’s recommendations (Catalog No. TD501-01/02, Vazyme).

Sequencing reads must undergo QC and adapter trimming to optimize the alignment process. FastQC (version 0.11.5) [45] was used to assess overall quality. Reads were trimmed for quality as well as the presence of adapter sequences using the Trim Galore Wrapper script [46] with default parameters. Raw ATAC-seq reads of sheep were mapped to the sheep reference genome [National Center for Biotechnology Information (NCBI) assembly Oar_v4.0] using Bowtie2 (version 2.2.8) [47] with default parameters. Duplicated reads were removed using the default parameters in Picard (version 2.1.1). Reads mapping to mitochondrial DNA were excluded from the analysis together with low-quality reads [Mapping Quality (MAPQ) < 20]. Then, accessible regions and narrow peaks were identified using MACS [48]. Open accessible peaks were identified in their biological replicates of each tissue using “bedtools intersect” parameter, and the consensus peak matrix with openness scores of each peak in each sample was constructed by merging these regions and calculating with the R package “Diffbind” (version 2.10.0) [49]. Finally, the genomic distributions of peaks were annotated using the R packages “GenomicFeatures”, “ChIPseeker”, and “AnnotationHub”.

### RNA-seq library preparation and sequencing

We prepared directional RNA-seq libraries from cells of the same samples used for ATAC-seq. One milliliter of TRIzol (Catalog No. 15596026, Invitrogen, Carlsbad, CA) was added to each sample and frozen at −80 °C until utilization. In all tissue samples collected for this study, total RNA was isolated from a frozen sample according to the TRIzol protocol (Catalog No. 15596026, Invitrogen). Sequencing libraries were generated using a NEBNext ultra RNA library prep kit for Illumina (Catalog No. E7760S, New England Biolabs, Ipswich, MA) according to the manufacturer’s recommendations. All prepared libraries were sequenced by the Illumina HiSeq X Ten platform, and paired-end reads with a length of 150 bp were generated. All sequencing procedures were performed by Novogene Technology (Beijing, China).

We obtained high-quality reads by removing adaptor sequences and filtering low-quality reads from raw reads using Trimmomatic (version 0.36) [50]. High-quality reads were all aligned to the NCBI assembly Oar_v4.0 reference sheep genome [51] by STAR (version 2.5.1) [52]. To improve the mapping rate, the unmapped reads of each sample were extracted by SAMtools (version 1.3) [53] for further mapping by HISAT2 (version 2.0.3-beta) [54]. We computed FPKM values for the genes in each sample using StringTie (version 1.3.4) [55].

As the samples were prepared and sequenced in three known distinct batches (see Table S1), we used the *removeBatchEffect()* function from the R *limma* package to build a linear model with the batch information and the cell types on log_2_ (FPKM + 1), and we regressed out the batch variable.

### Regulatory activity experiments

We selected fibroblast cells of ruminants for *in vitro* regulatory activity experiments. Sheep and goat fibroblast cells were provided by Guangxi University and were cultured in DMEM containing 10% fetal bovine serum (FBS; Catalog No. 10099141C, Gibco). All cell lines used in this study were maintained in the specified medium supplemented with 1× penicillin–streptomycin (Catalog No.15140122, Gibco) and incubated in 5% CO_2_ at 37 °C.

Firstly, sequences of candidate RSCNEs identified were cloned into pGL3-promoter vector (Catalog No. E1761, Promega, Madison, WI), which was digested by *Bam*HI and *Sal*I in the luciferase gene downstream. All constructs were further confirmed by sanger sequencing. Then, all reporter plasmid constructs were transfected using TurboFect (Catalog No. R0531, ThermoFisher Scientific, Waltham, MA), and renilla luciferase pRL-TK-Rluc (Catalog No. P1232, Promega) was used as control. Subsequently, luciferase expression was monitored with the dual luciferase assay (Catalog No. E1910, Promega) after 24-h transfection. Each assay was monitored at least five times, independently. The *t*-test was applied to calculate the significance of the regulatory activity using GraphPad Prism 7.0 software (Prism, San Diego, CA). Statistically significant differences need to meet the criterion of *P* < 0.05.

## Ethical statement

This study was carried out under the guidelines and approval of the Northwest A&F University Animal Care Committee (Approval No. NWAFAC1008).

## Code availability

All source codes are available freely for academic usage at https://ngdc.cncb.ac.cn/biocode/tools/BT007284.

## Data availability

Raw data from this study have been deposited in the NCBI (NCBI: PRJNA485657), which are publicly accessible at https://www.ncbi.nlm.nih.gov/, and also in the Genome Sequence Archive [56] at the National Genomics Data Center, Beijing Institute of Genomics, Chinese Academy of Sciences / China National Center for Bioinformation (GSA: CRA005494), which are publicly accessible at https://ngdc.cncb.ac.cn/gsa.

## Competing interests

The authors have declared no competing interests.

## CRediT authorship contribution statement

**Xiangyu Pan:** Conceptualization, Methodology, Software, Formal analysis, Investigation, Resources, Data curation, Writing – original draft, Visualization. **Zhaoxia Ma:** Methodology, Software, Formal analysis, Investigation, Writing – original draft, Visualization. **Xinqi Sun:** Methodology, Software, Formal analysis, Writing – original draft, Visualization. **Hui Li:** Validation, Resources. **Tingting Zhang:** Validation, Resources. **Chen Zhao:** Visualization. **Nini Wang:** Visualization. **Rasmus Heller:** Writing – review & editing. **Wing Hung Wong:** Supervision. **Wen Wang:** Conceptualization, Investigation, Supervision. **Yu Jiang:** Conceptualization, Investigation, Writing – review & editing, Supervision, Resources. **Yong Wang:** Conceptualization, Methodology, Investigation, Writing – review & editing, Supervision. All authors have read and approved the final manuscript.

## Supplementary material

Supplementary Table S1 Statistics of ATAC-seq data used in this study

Supplementary Table S2 Statistics of RNA-seq data used in our study

Supplementary Table S3 Identification of 1601 active-RSCNEs

Supplementary Table S4 Motif binding enrichment results of 1601 active-RSCNEs

Supplementary Table S5 Gene expression profile of 18 rumen TTFs during rumen and esophagus development

Supplementary Table S6 Upstream regulatory relationship of 18 rumen TTFs

Supplementary Table S7 Downstream regulatory relationship of 18 rumen TTFs

Supplementary Table S8 Differential regulatory sub-network between the rumen and esophagus

Supplementary Table S9 GO enrichment analysis of 52 TGs in the differential regulatory sub-network between the rumen and esophagus

Supplementary Table S10 Gene expression profile of 655 RSEGs represented by the FPKM value

## References

[1] Carroll S.B. (2008). Evo-devo and an expanding evolutionary synthesis: a genetic theory of morphological evolution. Cell.

[2] Pan X., Cai Y., Li Z., Chen X., Heller R., Wang N. (2021). Modes of genetic adaptations underlying functional innovations in the rumen. Sci China Life Sci.

[3] Smith J.J., Timoshevskaya N., Ye C., Holt C., Keinath M.C., Parker H.J. (2018). The sea lamprey germline genome provides insights into programmed genome rearrangement and vertebrate evolution. Nat Genet.

[4] Woolfe A., Goodson M., Goode D.K., Snell P., McEwen G.K., Vavouri T. (2005). Highly conserved non-coding sequences are associated with vertebrate development. PLoS Biol.

[5] Chen L., Qiu Q., Jiang Y., Wang K., Lin Z., Li Z. (2019). Large-scale ruminant genome sequencing provides insights into their evolution and distinct traits. Science.

[6] Wray G.A. (2007). The evolutionary significance of *cis*-regulatory mutations. Nat Rev Genet.

[7] Xiang R., Oddy V.H., Archibald A.L., Vercoe P.E., Dalrymple B.P. (2016). Epithelial, metabolic and innate immunity transcriptomic signatures differentiating the rumen from other sheep and mammalian gastrointestinal tract tissues. PeerJ.

[8] McLean C.Y., Bristor D., Hiller M., Clarke S.L., Schaar B.T., Lowe C.B. (2010). GREAT improves functional interpretation of *cis*-regulatory regions. Nat Biotechnol.

[9] Long H.K., Osterwalder M., Welsh I.C., Hansen K., Davies J., Liu Y.E. (2020). Loss of extreme long-range enhancers in human neural crest drives a craniofacial disorder. Cell Stem Cell.

[10] Duren Z., Chen X., Jiang R., Wang Y., Wong W.H. (2017). Modeling gene regulation from paired expression and chromatin accessibility data. Proc Natl Acad Sci U S A.

[11] Li L., Wang Y., Torkelson J.L., Shankar G., Pattison J.M., Zhen H.H. (2019). TFAP2C- and p63-dependent networks sequentially rearrange chromatin landscapes to drive human epidermal lineage commitment. Cell Stem Cell.

[12] Duren Z., Chen X., Xin J., Wang Y., Wong W.H. (2020). Time course regulatory analysis based on paired expression and chromatin accessibility data. Genome Res.

[13] Xin J., Zhang H., He Y., Duren Z., Bai C., Chen L. (2020). Chromatin accessibility landscape and regulatory network of high-altitude hypoxia adaptation. Nat Commun.

[14] Fath E.M., Schwarz R., Ali A.M. (1983). Micromorphological studies on the stomach of sheep during prenatal life. Anat Histol Embryol.

[15] Wardrop I.D. (1961). Some preliminary observations on the histological development of the fore-stomachs of the lamb I. Histological changes due to age in the period from 46 days of foetal life to 77 days of post-natal life. J Agric Sci.

[16] Irie N., Kuratani S. (2014). The developmental hourglass model: a predictor of the basic body plan?. Development.

[17] Cardoso-Moreira M., Halbert J., Valloton D., Velten B., Chen C., Shao Y. (2019). Gene expression across mammalian organ development. Nature.

[18] Gorkin D.U., Barozzi I., Zhao Y., Zhang Y., Huang H., Lee A.Y. (2020). An atlas of dynamic chromatin landscapes in mouse fetal development. Nature.

[19] Moore J.E., Purcaro M.J., Pratt H.E., Epstein C.B., Shoresh N., Adrian J. (2020). Expanded encyclopaedias of DNA elements in the human and mouse genomes. Nature.

[20] Visel A., Minovitsky S., Dubchak I., Pennacchio L.A. (2007). VISTA enhancer browser–a database of tissue-specific human enhancers. Nucleic Acids Res.

[21] Onimaru K. (2020). The evolutionary origin of developmental enhancers in vertebrates: insights from non-model species. Dev Growth Differ.

[22] Jonker L., Kist R., Aw A., Wappler I., Peters H. (2004). Pax9 is required for filiform papilla development and suppresses skin-specific differentiation of the mammalian tongue epithelium. Mech Dev.

[23] Manak J.R., Scott M.P. (1994). A class act: conservation of homeodomain protein functions. Dev Suppl.

[24] Takeuchi J.K., Koshiba-Takeuchi K., Matsumoto K., Vogel-Hopker A., Naitoh-Matsuo M., Ogura K. (1999). *Tbx5* and *Tbx4* genes determine the wing/leg identity of limb buds. Nature.

[25] Nair M., Teng A., Bilanchone V., Agrawal A., Li B., Dai X. (2006). Ovol1 regulates the growth arrest of embryonic epidermal progenitor cells and represses c-myc transcription. J Cell Biol.

[26] Koster M.I., Kim S., Mills A.A., DeMayo F.J., Roop D.R. (2004). p63 is the molecular switch for initiation of an epithelial stratification program. Genes Dev.

[27] Leask A., Byrne C., Fuchs E. (1991). Transcription factor AP2 and its role in epidermal-specific gene expression. Proc Natl Acad Sci U S A.

[28] Saito K., Michon F., Yamada A., Inuzuka H., Yamaguchi S., Fukumoto E. (2020). Sox21 regulates Anapc10 expression and determines the fate of ectodermal organ. iScience.

[29] Lee J., Rabbani C.C., Gao H., Steinhart M.R., Woodruff B.M., Pflum Z.E. (2020). Hair-bearing human skin generated entirely from pluripotent stem cells. Nature.

[30] Kim J.Y., Park M., Ohn J., Seong R.H., Chung J.H., Kim K.H. (2022). Twist2-driven chromatin remodeling governs the postnatal maturation of dermal fibroblasts. Cell Rep.

[31] Song Y., Zhang W., Zhang J., You Z., Hu T., Shao G. (2021). TWIST2 inhibits EMT and induces oxidative stress in lung cancer cells by regulating the FGF21-mediated AMPK/mTOR pathway. Exp Cell Res.

[32] Heinz S., Benner C., Spann N., Bertolino E., Lin Y.C., Laslo P. (2010). Simple combinations of lineage-determining transcription factors prime *cis*-regulatory elements required for macrophage and B cell identities. Mol Cell.

[33] Wilanowski T., Caddy J., Ting S.B., Hislop N.R., Cerruti L., Auden A. (2008). Perturbed desmosomal cadherin expression in grainy head-like 1-null mice. Embo J.

[34] Lynch V.J., Leclerc R.D., May G., Wagner G.P. (2011). Transposon-mediated rewiring of gene regulatory networks contributed to the evolution of pregnancy in mammals. Nat Genet.

[35] Ting C.N., Rosenberg M.P., Snow C.M., Samuelson L.C., Meisler M.H. (1992). Endogenous retroviral sequences are required for tissue-specific expression of a human salivary amylase gene. Genes Dev.

[36] Gregory T.R. (2008). The evolution of complex organs. Evo Edu Outreach.

[37] Griffith O.W., Wagner G.P. (2017). The placenta as a model for understanding the origin and evolution of vertebrate organs. Nat Ecol Evol.

[38] Lowe C.B., Kellis M., Siepel A., Raney B.J., Clamp M., Salama S.R. (2011). Three periods of regulatory innovation during vertebrate evolution. Science.

[39] Bejerano G., Pheasant M., Makunin I., Stephen S., Kent W.J., Mattick J.S. (2004). Ultraconserved elements in the human genome. Science.

[40] Belting H., Shashikant C., Ruddle F. (1998). Modification of expression and *cis*-regulation of *Hoxc8* in the evolution of diverged axial morphology. Proc Natl Acad Sci U S A.

[41] Zoonomia Consortium (2020). A comparative genomics multitool for scientific discovery and conservation. Nature.

[42] Feng S., Stiller J., Deng Y., Armstrong J., Fang Q., Reeve A.H. (2020). Dense sampling of bird diversity increases power of comparative genomics. Nature.

[43] Inoue J., Saitou N. (2021). dbCNS: a new database for conserved noncoding sequences. Mol Biol Evol.

[44] D'Alessio A.C., Fan Z.P., Wert K.J., Baranov P., Cohen M.A., Saini J.S. (2015). A systematic approach to identify candidate transcription factors that control cell identity. Stem Cell Rep.

[45] Andrews S. (2016).

[46] Krueger F. (2015).

[47] Langmead B., Salzberg S.L. (2012). Fast gapped-read alignment with Bowtie 2. Nat Methods.

[48] Zhang Y., Liu T., Meyer C.A., Eeckhoute J., Johnson D.S., Bernstein B.E. (2008). Model-based analysis of ChIP-seq (MACS). Genome Biol.

[49] Stark R., Brown G. (2011).

[50] Bolger A.M., Lohse M., Usadel B. (2014). Trimmomatic: a flexible trimmer for Illumina sequence data. Bioinformatics.

[51] Jiang Y., Xie M., Chen W., Talbot R., Maddox J.F., Faraut T. (2014). The sheep genome illuminates biology of the rumen and lipid metabolism. Science.

[52] Dobin A., Davis C.A., Schlesinger F., Drenkow J., Zaleski C., Jha S. (2013). STAR: ultrafast universal RNA-seq aligner. Bioinformatics.

[53] Li H., Handsaker B., Wysoker A., Fennell T., Ruan J., Homer N. (2009). The sequence alignment/map format and SAMtools. Bioinformatics.

[54] Kim D., Langmead B., Salzberg S.L. (2015). HISAT: a fast spliced aligner with low memory requirements. Nat Methods.

[55] Pertea M., Kim D., Pertea G.M., Leek J.T., Salzberg S.L. (2016). Transcript-level expression analysis of RNA-seq experiments with HISAT, StringTie and Ballgown. Nat Protoc.

[56] Chen T., Chen X., Zhang S., Zhu J., Tang B., Wang A. (2021). The Genome Sequence Archive Family: toward explosive data growth and diverse data types. Genomics Proteomics Bioinformatics.

